# Comparative analysis of the effects of OLIF and TLIF on adjacent segments after treatment of L4 degenerative lumbar spondylolisthesis

**DOI:** 10.1186/s13018-022-03084-7

**Published:** 2022-04-04

**Authors:** Guang-qing Li, Tong Tong, Lin-feng Wang

**Affiliations:** Spine Surgery Department 1, The Third Hospital of Hebei Medical University, Shijiazhuang, China

**Keywords:** Oblique lumbar interbody fusion, Transforaminal lumbar interbody fusion, Degenerative lumbar spondylolisthesis, Adjacent segmental degeneration

## Abstract

**Background:**

The fusion of the lumbar spine may lead to the degeneration of the adjacent segments. In this study, the effects of OLIF and TLIF on adjacent segments after treatment of L4 degenerative lumbar spondylolisthesis (DLS) were compared and analysed.

**Methods:**

This was a retrospective analysis of the medical records of consecutive patients treated with OLIF or TLIF for L4DLS. They were divided into the OLIF group and TLIF group based on different treatment methods. Cage height, segmental lordosis (SL), lumbar lordosis (LL), pelvic incidence (PI), pelvic tilt (PT), and sacral slope (SS) were compared between the two groups, and the postoperative biomechanical changes were analysed by establishing the disc angle (DA). The clinical outcomes were analysed by comparing the visual analogue scale (VAS), Oswestry Disability Index (ODI) and incidence of adjacent segment disease (ASDis) between the two groups. The intervertebral disc height (IDH), intervertebral foramen height (IDH), intervertebral foramen area (IFA), sliding distance (SD), and angular displacement (AD) in L3-4 and L5-S1 were compared between the two groups. The incidence of aggravated disc degeneration (ADD), the incidence of aggravated zygapophyseal joint degeneration (AJD) and the incidence of adjacent segment degeneration (ASDeg) were compared between the two groups for radiological degeneration.

**Results:**

At the last follow-up, there was one case of ASDis in the OLIF group (2.78%) and two cases in the TLIF group (5.56%). At the last follow-up, compared with the preoperative values, IDH, IFH, and IFA of the adjacent segments above and below L4-5 decreased in both groups (*P* < 0.05); the SD and AD increased in both groups (*P* < 0.05). The cage height and L4-5 IDH in the OLIF group were significantly higher than those in the TLIF group (*P* < 0.05). SL, LL, PT, SS, and L5- S1DA were significantly improved in the OLIF group compared with the TLIF group (*P* < 0.05). The incidence of L3-4ASDeg in the two groups was higher than that of L5-S1. The incidence of ASDeg and the incidence of L5-S1ADD in the OLIF group were lower than those in the TLIF group, but the incidence of L5-S1AJD was higher than that in the TLIF group.

**Conclusion:**

L4DLS after OLIF and TLIF treatment will cause adjacent segment degeneration, and L3-4 degeneration is more obvious than L5-S1 degeneration. OLIF has more advantages in restoring lumbar sagittal balance. Compared with TLIF, OLIF can weaken the degeneration of the L5-S1 disc and increase the degeneration of the L5-S1 zygapophyseal joints.

**Supplementary Information:**

The online version contains supplementary material available at 10.1186/s13018-022-03084-7.

## Background

Degenerative lumbar spondylolisthesis (DLS) refers to the slip of the upper vertebral body relative to the lower vertebral body on the basis of degeneration, without defects in the vertebral arch [1]. With the development of surgical techniques, DLS has evolved from previous zygapophyseal joint fusion to interbody fusion represented by transforaminal lumbar interbody fusion (TLIF) to minimally invasive interbody fusion represented by oblique lumbar interbody fusion (OLIF). However, spinal fusion may bring about degeneration of adjacent segments above and below the fusion area [2, 3]. Many scholars confuse the concepts of adjacent segment degeneration and adjacent segment disease. Adjacent segment degeneration (ASDeg) is defined as radiographic changes seen at levels adjacent to a previous spinal fusion procedure that do not necessarily correlate with any clinical findings [2]. Adjacent segment disease (ASDis) represents symptomatic adjacent segment degeneration and causes pain or numbness due to postoperative spinal instability or nerve compression at the same level [3]. The reported ASDeg rates in the literature range from 2.6 to 30.3% [4, 5]. Known risk factors associated with the development of ASDeg are age, sex, obesity, pre-existing degeneration, number of fused segments, and reduction in postoperative lumbar lordosis [6, 7]. It is beneficial to reduce the occurrence of ASDeg by restoring the height of the surgical segmental intervertebral space and segmental lordosis to restore lumbar lordosis (LL) and correct sagittal imbalance. However, some scholars believe that surgical treatment of single-segment lumbar disease has little effect on restoring lumbar lordosis. It is unclear whether different surgical approaches or fusion methods produce different effects on the adjacent segments.

This study aimed to determine whether lumbar lordosis and other spinal-pelvic parameters can be improved after L4DLS is treated with OLIF or TLIF. We analysed whether the effects of OLIF and TLIF on the adjacent segments above and below L4-5 differ in clinical and imaging presentation.

## Materials and methods

This paper was prepared in accordance with the STROBE checklist (Additional file 1). A total of 613 medical records of consecutive patients treated with OLIF or TLIF for lumbar spondylolisthesis in the Third Hospital of Hebei Medical University from January 2015 to June 2020 were retrospectively analysed. The inclusion criteria were as follows: (1) L4 degenerative lumbar spondylolisthesis (Meyerding classification grade [8] is I); (2) treatment with OLIF or TLIF surgery; (3) LL < 40°; (4) complete follow-up data; and (5) follow-up time no less than 40 months. The exclusion criteria were as follows: (1) isthmic lumbar spondylolisthesis, pathological lumbar spondylolisthesis, traumatic lumbar spondylolisthesis, etc.; (2) spinal tumour, spinal deformity, spinal infection, etc.; (3) previous history of lumbar surgery; and (4) incomplete follow-up data. Patients were divided into the OLIF group and TLIF group based on the different surgical methods. In line with the above inclusion and exclusion criteria, 36 patients in the OLIF group and 36 patients in the TLIF group were included in the study. The study was approved by the Ethics Committee of the Third Hospital of Hebei Medical University. All selected subjects provided written informed consent to participate.

### Surgical technique

#### OLIF

The patient was placed in the right lateral position after satisfactory general anaesthesia. Blunt separation of the external oblique abdominis, internal oblique abdominis and transversus abdominis muscles was performed. The L4-5 vertebral space was entered between the abdominal aorta and the psoas major muscle. The intervertebral disc annulus fibrosus was cut, and the intervertebral disc tissue and cartilage endplate were completely scraped. An appropriately sized cage filled with allogeneic bone was inserted into the disc space. The patient was placed in the prone position. Four pedicle screws were inserted using the Wiltse approach. After installing the connecting bars and locking them with pressure, segmental lordosis was further restored.

#### TLIF

The patient was placed in the prone position after satisfactory general anaesthesia. Four pedicle screws were inserted into the pedicle via a posterior midline approach. The nerve root on the symptomatic side was decompressed, and the hyperplastic bone and ligamentum flavum were removed. The intervertebral disc tissue and cartilage endplates were scraped via the intervertebral foramen. An appropriate-sized cage filled with autologous bone was placed in the intervertebral space via the intervertebral foramen. Then, the connecting bars were installed and locked with pressure. According to Umile Giuseppe Longo et al. [9], patients with lumbar spondylolisthesis who undergo reduction and fixation fusion and in situ fixation fusion both have good clinical results, with no statistically significant difference between the two modalities. Herein, we only reduced unbalanced-pelvis spondylolisthesis.

### Evaluating indicators

#### Basic patient information

The basic data, such as sex, age, body mass index and follow-up time, were compared between the two groups, and the cage height was compared between the two groups.

#### Clinical analyses

The visual analogue scale (VAS) was used to evaluate low back pain (VAS-B) and leg pain (VAS-L), and the Oswestry Disability Index (ODI) and the incidence of ASDis were used to evaluate the clinical outcomes of the two groups. ASDis defined as the reappearance of lower extremity radiating pain, lower extremity numbness, intermittent claudication or even cauda equina syndrome consistent with ASDeg after lumbar fusion [10].

### Radiographic analyses

All radiological parameters were measured by a radiologist and a spine surgeon and averaged. The changes of L4-5intervertebral disc height (IDH), segmental lordosis (SL), lumbar lordosis (LL), pelvic incidence (PI), pelvic tilt (PT) and sacral slope (SS) in the sagittal position were compared between the two groups at preoperation and final follow-up. The parameters of L3-4 and L5-S1 in IDH, intervertebral foramen height (IDH), intervertebral foramen area (IFA), sliding distance (SD), angular displacement (AD) and disc angle (DA) were compared between the two groups. The incidence of aggravated disc degeneration (ADD), incidence of aggravated zygapophyseal joint degeneration (AJD) and incidence of ASDeg were compared between the two groups at the last follow-up.

IDH was defined as the average anterior disc height and posterior disc height on the lumbar lateral X-ray. SL was defaulted to L4-5SL in this study, which was defined as the angle formed between the upper endplate of L4 and the lower endplate of L5 on the lumbar lateral X-ray. SD was defined as the sliding distance between the upper vertebral body and the lower vertebral body on the lumbar lateral X-ray. AD was defined as the angle of change in the angle consisting of the inferior endplate of the superior vertebral body and the superior endplate of the inferior vertebral body on the lumbar hyperextension–hyperflexion position X-ray. Since the upper and lower endplates constituting the intervertebral disc are not in the same plane, resulting in a large anterior angle and small posterior angle, DA was established to evaluate the changes before and after the operation. DA was defined as the angle between the median line of the intervertebral disc and the horizontal line on the lateral lumbar X-ray. According to the Pfirrmann grading standard [11], at the last follow-up relative to preoperative, ADD was defined as ≥ 1 grade. According to the Pathria grading standard [12], at the last follow-up relative to preoperative, AJD was defined as ≥ 1 grade. Based on a previous report [5, 13, 14], ASDeg was defined as follows: (1) disc degeneration, such as loss of disc height of more than 10%; (2) listhesis (anterolisthesis, retrolisthesis) of more than 4 mm; (3) angle change greater than 10 degrees between adjacent bodies on flexion and extension radiographs; (4) occurrence of symptomatic disc herniation or spinal stenosis confirmed by magnetic resonance imaging (MRI); (5) hypertrophic facet arthropathy; (6) osteophyte formation of more than 3 mm; (7) scoliosis; and (8) compression fracture.

### Statistical analysis

All statistical analyses were performed using SPSS software. (Version 26.0, Chicago, IL, the USA). The concordance between the two raters was examined using the Kappa concordance test. The Kolmogorov–Smirnov test was used to examine the normal distribution within the group. For continuous variables that conformed to a normal distribution, the two-sample t-test was used for comparisons between groups. The paired t-test was used to compare the values of the variables between preoperation and the last follow-up. For continuous variables that did not conform to a normal distribution, the Mann–Whitney U-test was used for comparisons between groups. The Wilcoxon rank-sum test was used to compare the values of the variables between preoperation and the last follow-up. The chi-square Fisher's exact test was used to compare the categorical variables between the groups. Multivariate linear regression was used to examine the associations between DA and several variables. *P* < 0.05 was considered statistically significant.

## Results

The cage height of the OLIF group was significantly higher than that of the TLIF group, and the difference was statistically significant (*P* < 0.05). There was no statistical significance (*P* > 0.05) between the two groups in terms of sex, age, body mass index and follow-up time (Table 1). Furthermore, interobserver agreement was excellent for both readers, with kappa values ranging from 0.82 to 0.90.

**Table 1:** Comparison of baseline patients data

	OLIF group (*n* = 36)	TLIF group (*n* = 36)	*P* value
Gender (M/F)	6/30	10/26	0.396
Age (years)	58.52 ± 7.26	59.88 ± 7.04	0.629
Body mass index (kg/m^2^)	25.16 ± 2.66	24.87 ± 2.42	0.713
Follow-up time (months)	43.13 ± 3.24	44.42 ± 4.54	0.932
Cage height (mm)	12.08 ± 0.84	9.97 ± 1.06	< 0.001

**Fig. 1:**
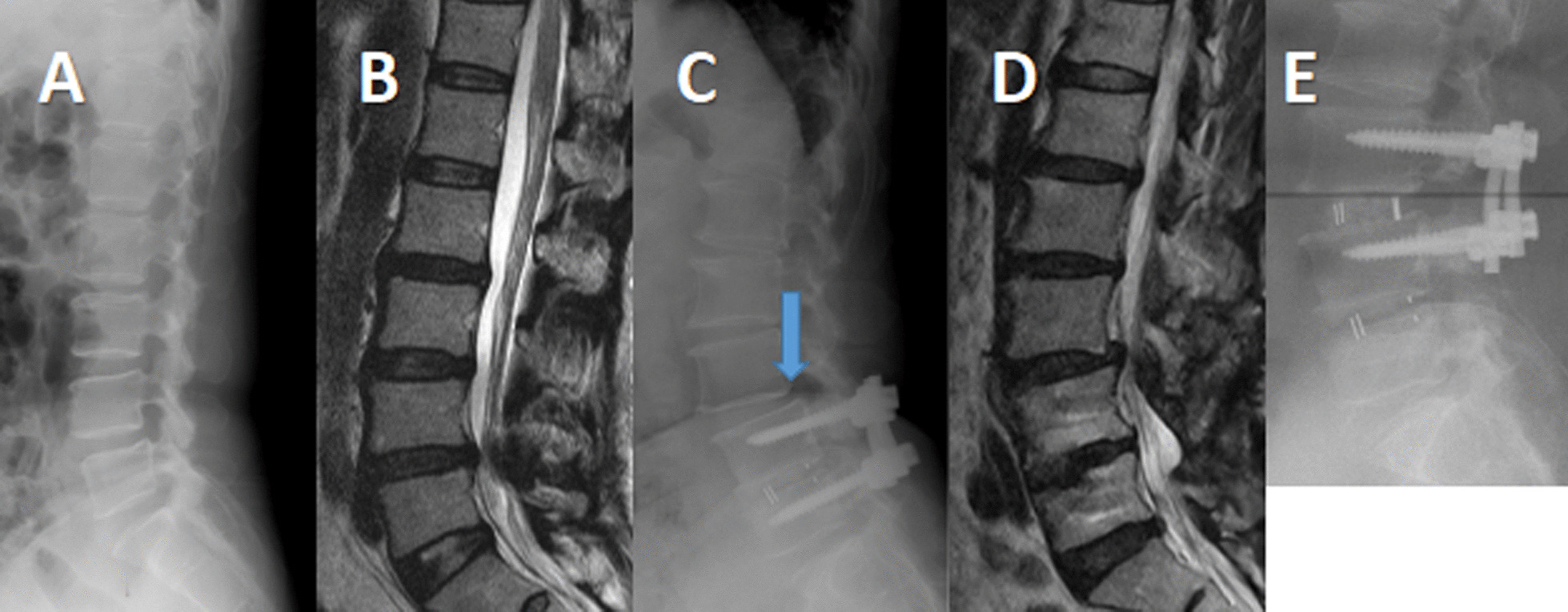
**A**, **B** Lateral X-ray and sagittal magnetic resonance image of lumbar in a 48-year-old female patient complaining of lumbar pain with right lower extremity pain. It showed L4 degenerative lumbar spondylolisthesis and L4-5 disc degeneration. **C**, **D** 47 months after TLIF, this patient again complained of low back pain with numbness in both lower extremities. Lateral X-ray and sagittal magnetic resonance image of lumbar showed L3 spondylolisthesis and L3-4, L5-S1 intervertebral disc degeneration aggravated relative to preoperative. **E** The patient selected TLIF revision surgery. Lateral X-ray of lumbar spine showed satisfactory L3 reduction

At the last follow-up, VAS and ODI scores decreased significantly in the two groups compared with preoperative values (*P* < 0.05). VAS and ODI scores were not statistically significant in the two groups at preoperation and final follow-up (*P* > 0.05). One patient in the OLIF group developed ASD at the final follow-up, and the incidence of ASD was 2.78% (Table 2); this patient chose conservative treatment. Two patients in the TLIF group developed ASDis, and the incidence of ASDis was 5.56%. One patient chose conservative treatment, and the other patient chose TLIF revision surgery (Fig. 1).

**Table 2:** Clinical outcomes of OLIF group and TLIF group

	OLIF group (*n* = 36)	TLIF group (*n* = 36)	*P* value
VAS-B			
Preoperative	6.89 ± 2.01	7.01 ± 1.98	0.684
At the last follow-up	1.84 ± 0.71	2.03 ± 0.93	0.258
VAS-L			
Preoperative	8.42 ± 1.50	8.62 ± 1.39	0.925
At the last follow-up	1.90 ± 0.89	2.05 ± 0.92	0.525
ODI			
Preoperative	68.58 ± 8.18	66.32 ± 10.42	0.602
At the last follow-up	10.54 ± 5.39	12.66 ± 6.83	0.452
Incidence of ASDis at the last follow-up	1/36 (2.78%)	2/36 (5.56%)	1.000

In terms of spine-pelvis sagittal parameters, L4-5IDH, SL, LL, PT and SS improved in both groups at the last follow-up compared to the preoperative values, but the improvement was more pronounced in the OLIF group (*P* < 0.05). There was no significant difference in L4-5 IDH, SL, LL, PT or SS between the two groups before surgery (*P* > 0.05), but at the final follow-up, the OLIF group improved significantly compared with the TLIF group (*P* < 0.05). There was no significant change in PI at the last follow-up between the two groups compared to the preoperative PI (*P* > 0.05), and the difference between the two groups was not statistically significant (Table 3).

**Table 3:** Comparison of spine-pelvis sagittal parameters

	OLIF group (*n* = 36)	TLIF group (*n* = 36)	*P* value
L4-5IDH (mm)			
Preoperative	8.49 ± 1.76	8.47 ± 1.66	0.458
At the last follow-up	11.72 ± 1.87	9.26 ± 1.19	< 0.001
SL (°)			
Preoperative	11.14 ± 4.74	10.57 ± 5.74	0.873
At the last follow-up	14.86 ± 5.58	12.11 ± 3.51	0.041
LL (°)			
Preoperative	33.25 ± 5.34	33.67 ± 7.51	0.247
At the last follow-up	42.30 ± 6.27	34.09 ± 9.74	0.035
PI (°)			
Preoperative	51.42 ± 9.48	50.66 ± 10.47	0.304
At the last follow-up	52.34 ± 8.90	51.53 ± 7.96	0.356
PT (°)			
Preoperative	24.49 ± 6.02	23.43 ± 5.48	0.652
At the last follow-up	16.52 ± 4.21	21.78 ± 3.75	0.042
SS (°)			
Preoperative	28.43 ± 5.74	27.67 ± 2.08	0.652
At the last follow-up	36.96 ± 7.17	30.98 ± 5.30	0.039

In L3-4, the IDH, IFH and IFA of the two groups at the last follow-up were decreased compared with those before the operation (*P* < 0.05), and the SD and AD were increased compared with those before the operation (*P* < 0.05). At the last follow-up, DA in both groups was higher than before the operation, but the difference was not statistically significant (*P* > 0.05). There was no statistically significant difference in IDH, IFH, IFA, SD, AD or DA between the two groups at the time points of preoperation and final follow-up (Table 4).

**Table 4:** (L3-4)Radiological outcomes of OLIF group and TLIF group

	OLIF group (*n* = 36)	TLIF group (*n* = 36)	*P* value
L3-4IDH (mm)			
Preoperative	10.13 ± 1.52	10.68 ± 0.94	0.419
At the last follow-up	9.46 ± 1.73	9.85 ± 1.01	0.499
L3-4IFH (mm)			
Preoperative	20.58 ± 3.84	21.84 ± 3.46	0.500
At the last follow-up	19.50 ± 3.84	20.06 ± 2.78	0.591
L3-4IFA (mm^2^)			
Preoperative	193.47 ± 32.68	220.44 ± 49.28	0.124
At the last follow-up	173.47 ± 29.18	191.71 ± 37.67	0.179
L3-4SD (mm)			
Preoperative	1.74 ± 0.40	1.71 ± 0.31	0.633
At the last follow-up	2.10 ± 0.73	2.28 ± 0.86	0.583
L3-4AD (°)			
Preoperative	3.44 ± 2.19	3.95 ± 2.40	0.652
At the last follow-up	4.53 ± 2.91	4.75 ± 2.09	0.855
L3-4DA (°)			
Preoperative	5.21 ± 3.60	5.00 ± 3.69	0.476
At the last follow-up	7.98 ± 5.51	6.19 ± 2.38	0.897

In L5-S1, the IDH, IFH and IFA of the two groups at the last follow-up were decreased compared with those before the operation (*P* < 0.05), and the SD and AD were increased compared with those before the operation (*P* < 0.05). In both groups, the DA increased at the last follow-up compared with the preoperative period, but the increase was significant in the OLIF group (*P* < 0.05) and not in the TLIF group (*P* > 0.05). At the last follow-up, DA in the OLIF group was significantly higher than that in the TLIF group (*P* < 0.05). There was no statistically significant difference in IDH, IFH, IFA, SD or AD between the two groups at the time points of preoperation and final follow-up (Table 5).

**Table 5:** (L5-S1)Radiological outcomes of OLIF group and TLIF group

	OLIF group (*n* = 36)	TLIF group (*n* = 36)	*P* value
L5-S1IDH (mm)			
Preoperative	10.89 ± 2.17	11.43 ± 1.65	0.522
At the last follow-up	10.27 ± 1.87	10.59 ± 1.76	0.967
L5-S1IFH (mm)			
Preoperative	16.06 ± 2.64	16.25 ± 3.73	0.548
At the last follow-up	14.95 ± 2.55	15.63 ± 3.52	0.380
L5-S1IFA (mm^2^)			
Preoperative	125.60 ± 42.86	132.13 ± 37.27	0.689
At the last follow-up	112.95 ± 37.49	123.47 ± 34.05	0.467
L5-S1SD (mm)			
Preoperative	1.68 ± 0.31	1.71 ± 0.36	0.705
At the last follow-up	1.95 ± 0.66	1.90 ± 0.74	0.632
L5-S1AD (°)			
Preoperative	5.05 ± 3.63	4.71 ± 3.50	0.860
At the last follow-up	6.38 ± 3.39	5.92 ± 3.52	0.482
L5-S1DA (°)			
Preoperative	25.13 ± 8.35	24.33 ± 4.62	0.524
At the last follow-up	33.60 ± 4.53	26.98 ± 7.68	0.047

In the OLIF group, the incidences of ADD in L3-4, L3-4 & L5-S1, L5-S1 and the total were 19.44%, 5.56%, 8.33% and 33.33%, respectively. In the TLIF group, the incidences of ADD in L3-4, L3-4 & L5-S1, L5-S1 and the total were 25%, 8.33%, 16.67% and 50%, respectively. In the OLIF group, the incidences of AJD in L3-4, L3-4 & L5-S1, L5-S1 and the total were 38.89%, 22.22%, 22.22% and 83.33%, respectively. In the TLIF group, the incidences of AJD in L3-4, L3-4 & L5-S1, L5-S1 and the total were 41.67%, 22.22%, 13.89% and 77.78%, respectively. In the OLIF group, the incidences of ASDeg in L3-4, L3-4 & L5-S1, L5-S1 and the total were 11.11%, 2.78%, 5.56% and 19.44%, respectively. In the TLIF group, the incidences of ASDeg in L3-4, L3-4 & L5-S1, L5-S1 and the total were 16.67%, 2.78%, 8.33% and 27.78%, respectively (Table 6).

**Table 6:** Degeneration comparison of OLIF group and TLIF group

	OLIF group (*n* = 36)	TLIF group (*n* = 36)	*P* value
Incidence of ADD			
L3-4	7/36 (19.44%)	9/36 (25%)	0.778
L3-4 & L5-S1	2/36 (5.56%)	3/36 (8.33%)	1.000
L5-S1	3/36 (8.33%)	6/36 (16.67%)	0.478
Total	12/36 (33.33%)	18/36 (50%)	0.232
Incidence of AJD			
L3-4	14/36 (38.89%)	15/36 (41.67%)	0.500
L3-4 & L5-S1	8/36 (22.22%)	8/36 (22.22%)	1.000
L5-S1	8/36 (22.22%)	5/36 (13.89%)	0.541
Total	30/36 (83.33%)	28/36 (77.78%)	0.767
Incidence of ASDeg			
L3-4	4/36 (11.11%)	6/36 (16.67%)	0.735
L3-4 & L5-S1	1/36 (2.78%)	1/36 (2.78%)	1.000
L5-S1	2/36 (5.56%)	3/36 (8.33%)	1.000
Total	7/36 (19.44%)	10/36 (27.78%)	0.580

Multiple regression analysis showed that L5-S1DA increased with increasing L4-5IDH and increased with decreasing PT. The regression model was L5-S1DA = 0.582 × L4-5IDH-0.404 × PT + 33.382 [*P* < 0.05, *R*^2^ = 49%].

## Discussion

Lumbar fusion is the most common treatment for degenerative lumbar spondylolisthesis. From the earliest posterolateral lumbar fusion, a variety of spinal fusion surgeries gradually emerged. Because TLIF can achieve interbody fusion and immediate segmental stability while achieving nerve decompression, it has gradually become the mainstream surgical method for the treatment of degenerative lumbar spondylolisthesis. As OLIF can avoid bone destruction and does not invade the spinal canal, it gradually rises in clinical practice [15, 16]. The presence of bone bridges between two contiguous vertebrae as pathognomonic criteria for anterior fusion. The disc and the zygapophyseal joints constitute an articular complex, and zygapophyseal joints ankylosis represents facets joints fusion. The formation of intervertebral bone bridges and complete ankylosis of the zygapophyseal joints represents the realization of true spinal fusion [17]. True spinal fusion reduces the mechanical stress on the instrumentation system and thus the risk for its failure. True spinal fusion can better reduce pain scores and dysfunction than partial spinal fusion. Min Cheol Chang et al. [18] conducted a systematic review and found no significant difference in clinical and radiological results between OLIF and TLIF in the treatment of lumbar spondylolisthesis. Ai-Feng Liu et al. [19] believed that OLIF and TLIF had no significant difference in relieving pain and improving function, but OLIF could improve LL and IDH compared with TLIF. Minimally invasive surgery (such as MIS-TLIF) has been shown to reduce intraoperative blood loss, the incidence of perioperative infection, and the overall hospital stay and early return to daily life compared with open TLIF [20]. However, whether MIS-TLIF has the advantage of reducing ASD compared with TLIF remains controversial. XLIF also does not invade the spinal canal, avoiding damage to the bone structure. However, XILF increases the potential for injury to the psoas major and lumbar plexus nerves relative to OLIF. Due to the different surgical methods of OLIF and TLIF, as well as the different radiological results, whether the impact of fusion on adjacent segments is the same is still unclear. Although Kotani et al. [21] compared the effects of OLIF and MIS-TLIF on adjacent segments, Wang et al. [22] compared the effects of OLIF and TLIF on adjacent segments in biomechanics. However, the studies conducted by the above and others are not particularly detailed. This is the first study to conduct a relatively detailed analysis of the impact of TLIF and OLIF on adjacent segments in the operation of degenerative lumbar diseases.

This study showed that OLIF and TLIF were similar in terms of clinical outcomes at the time of the final follow-up. Although the incidence of ASDis in the OLIF group was lower than that in the TLIF group, there was no significant difference between the two groups. In terms of sagittal balance, L4-5 IDH, SL, LL, PT and SS improved better in the OLIF group than in the TLIF group, which was similar to the findings of Li et al. [23]. In L3-4, the IDH, IFH and IFA in the two groups decreased compared with those before the operation, while SD, AD and DA increased compared with those before the operation. In the OLIF group, the L5-S1DA was significantly different compared to the TLIF group. The incidence of ADD and ASDeg in the OLIF group was lower than that in the TLIF group. The incidence of L3-4ADD was higher than that of L5-S1. In terms of the incidence of AJD, the incidence of L5-S1 and the overall incidence in the OLIF group were higher than those in the TLIF group.

Patients with degenerative lumbar spondylolisthesis have changes in their spine-pelvis sagittal parameters, and to compensate for the sagittal imbalance due to degenerative changes, there is a decrease in SS and an increase in PT. Kim et al. [24] concluded that the posterior tilting of the pelvis due to high PT causes the lumbosacral muscles and ligaments to be under high tension, which is not conducive to the relief of postoperative lumbosacral pain and will also accelerate the degeneration of the adjacent segments. Phillipsren et al. [25] examined the sagittal imbalance after lumbar degeneration and found that in order to compensate for the sagittal imbalance in the human body, sacral anteversion occurs, and SS and LL decreased simultaneously, thus increasing the hip and knee joint mechanical burden and aggravating the symptoms of low back and leg pain. Djurasovic et al. [26] confirmed that a decrease in SL and LL increases the risk of ASD. The current study also showed similar results. Compared with the TLIF group, the OLIF group has more advantages in restoring SL, LL, PT and SS. Combined with the fact that the incidences of ASDeg and ASDis were lower in the OLIF group than in the TLIF group, it can be confirmed that correction of sagittal imbalance can reduce the incidence of ASD.

**Fig. 2:**
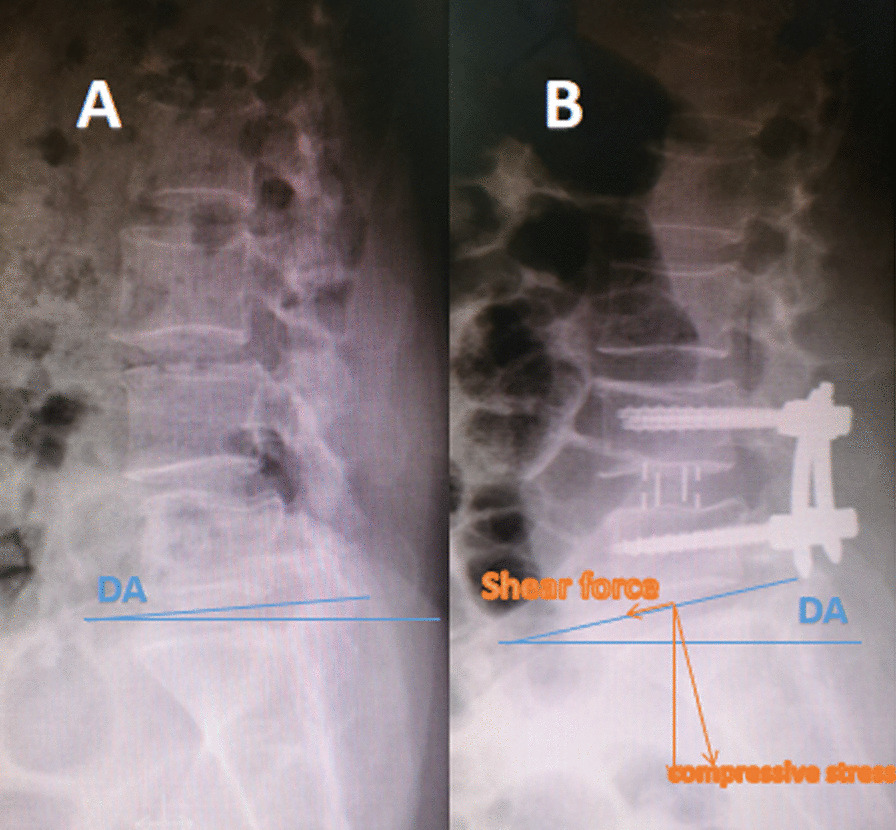
**A** Preoperative DA was 3.15°. **B** After OLIF, DA was 10.49°. Shear force = lumbar gravity × sinDA, compressive stress = lumbar gravity × cosDA

The occurrence of ASD is associated with postoperative biomechanical changes. However, how exactly the biomechanics is changed, numerous scholars have different answers. The novelty of this study is the establishment of DA to explain the postoperative biomechanical changes. The gravity borne by the lumbar spine can be divided into the force perpendicular to the intervertebral disc median line and the force parallel to the intervertebral disc median line. The force perpendicular to the midline of the intervertebral disc is compressive stress, and the force parallel to the midline of the intervertebral disc is shear stress. According to physical and mathematical principles, compressive stress = lumbar gravity × cosDA, DA increases, and the compressive stress of the intervertebral disc decreases. Shear force = lumbar gravity × sinDA, DA increases, and the shear force of the intervertebral disc increases (Fig. 2). At the final follow-up, L5-S1DA was significantly different in the OLIF group relative to the TLIF group. In L5-S1, the compressive stress of OLIF on the L5-S1 intervertebral disc decreased by 5.83 N compared with that of TLIF, and the shear stress increased by 9.97 N. The decrease in compressive stress will weaken the degeneration of the intervertebral disc, and the increase in shear stress will accelerate the degeneration of zygapophyseal joints. This was consistent with the fact that the incidence of L5-S1ADD in the OLIF group was lower than that in the TLIF group, and the incidence of L5-S1AJD was higher than that in the TLIF group. Multiple regression analysis showed that the L5-S1DA increase was linearly related to the L4-5IDH increase and PT decrease. Single-factor analysis showed that L4-5IDH in the OLIF group was significantly higher than that in the TLIF group, which led to an increase in L5-S1DA, which weakened L5-S1 intervertebral disc degeneration and increased L5-S1 zygapophyseal joint degeneration. We call this the overstrengthening effect.

This study has several limitations. First, the sample size was small, and the follow-up time was not long. In the future, clinical observation with multiple centres, a large sample size, and long-term follow-up are necessary to evaluate the impact of OLIF and TLIF on adjacent segments. Second, the biomechanical changes due to the overstrengthening effect shown in this study were not subjected to biomechanical experiments. Last but not least, this study is a retrospective case–control study, and higher-level evidence is required in the future.

## Conclusions

After OLIF and TLIF treatment of L4 degenerative spondylolisthesis, adjacent segment degeneration will occur. OLIF is more advantageous in restoring sagittal balance. Compared with TLIF, OLIF can weaken the degeneration of the L5-S1 intervertebral disc and increase the degeneration of the L5-S1 zygapophyseal joints.

## Supplementary Information

**Additional file 1.** STROBE Statement-checklist of items of observational studies.

## Data Availability

The data that support the findings of this study are available on request from the corresponding author. The data are not publicly available due to privacy or ethical restrictions.

## Abbreviations

DLS: Degenerative lumbar spondylolisthesis
TLIF: Transforaminal lumbar interbody fusion
OLIF: Oblique lumbar interbody fusion
ASDeg: Adjacent segment degeneration
ASDis: Adjacent segment disease
VAS: Visual analogue scale
VAS-B: VAS for low back pain
VAS-L: VAS for leg pain
ODI: Oswestry Disability Index
IDH: Intervertebral disc height
SL: Segmental lordosis
LL: Lumbar lordosis
PI: Pelvic incidence
PT: Pelvic tilt
SS: Sacral slope
IFH: Intervertebral foramen height
IFA: Intervertebral foramen area
SD: Sliding distance
AD: Angular displacement
DA: Disc angle
ADD: Aggravated disc degeneration
AJD: Aggravated zygapophyseal joints degeneration
L3-4 & L5-S1: L3-4 and L5-S1
